# Epidemiology, clinical presentation, treatment, and outcome of neurosarcoidosis: A mono-centric retrospective study and literature review

**DOI:** 10.3389/fneur.2022.970168

**Published:** 2022-10-25

**Authors:** Pauline Sambon, Amina Sellimi, Alexandra Kozyreff, Olivier Gheysens, Lucie Pothen, Halil Yildiz, Vincent van Pesch

**Affiliations:** ^1^Department of Internal Medicine and Infectious Diseases, Cliniques Universitaires Saint-Luc, Université Catholique de Louvain, Brussels, Belgium; ^2^Department of Neurology, Cliniques Universitaires Saint-Luc, Université Catholique de Louvain, Brussels, Belgium; ^3^Department of Ophthalmology, Cliniques Universitaires Saint-Luc, Université Catholique de Louvain, Brussels, Belgium; ^4^Department of Nuclear Medicine, Cliniques Universitaires Saint-Luc and Institute of Clinical and Experimental Research (IREC), Université Catholique de Louvain, Brussels, Belgium

**Keywords:** sarcoidosis, neurosarcoidosis, methotrexate, azathioprine, TNF-α antagonist, outcome

## Abstract

**Introduction:**

Neurosarcoidosis is a rare granulomatous disorder, and treatment guidelines are mainly based on retrospective studies.

**Materials and methods:**

This retrospective study was performed to provide a detailed description of the clinical characteristics and treatment outcomes of patients with neurosarcoidosis followed at Cliniques Universitaires Saint Luc in Belgium. The second objective of our study was to perform a comparative literature review of neurosarcoidosis, with a focus on treatment outcomes with the use of TNF-α antagonist.

**Results:**

Among 180 patients with sarcoidosis followed in our hospital, 22 patients with neurosarcoidosis were included in the final analysis. Our literature research identified 776 articles of which 35 articles met our inclusion criteria, including 1,793 patients diagnosed with neurosarcoidosis. In our cohort, the majority of patients (86%) were diagnosed with systemic sarcoidosis which was similar to that reported in the literature (83%). Serum CRP and calcemia were elevated only in 33 and 18% of patients, respectively. Serum lysozyme and angiotensin-converting enzyme were elevated in 79 and 16% of patients, respectively. Lumbar puncture and CSF fluid analysis were performed in 15/22 patients and were abnormal in all patients. Brain MRI was performed in 21/22 patients and showed abnormalities in 16 patients consisting of parenchymal lesions in 63%, hypothalamic-pituitary axis lesions in 38%, and meningeal enhancement in 31%. In both cohort patients, methotrexate was the most frequently used treatment (>45% of cases) with a favorable outcome in an average of 50% of patients. A TNF-α antagonist was administered in 9% of patients in our cohort and in 27% of patients in the literature review. The proportion of favorable outcomes in literature research was significantly higher in patients treated with TNF-α antagonists compared to methotrexate (*p* < 0.0001), mycophenolate mofetil (*p* < 0.0001), or azathioprine (*p* < 0.0001).

**Conclusion:**

The results of our cohort and literature review confirm that neurosarcoidosis occurred most frequently in the context of systemic sarcoidosis. Methotrexate is the most frequent second-line therapy. The effectiveness of therapy with TNF-α antagonists is well-demonstrated and associated with a better outcome. Their earlier use during the disease course among aggressive and/or refractory neurosarcoidosis should be considered.

## Introduction

Sarcoidosis is a systemic inflammatory disorder characterized by non-caseating granulomatous lesions. Although all organs may be affected, it occurs most frequently (>90% of cases) in lymph nodes, particularly mediastinal; but also in lungs, skin, and eyes (e.g., uveitis) (1). Skin involvement (lupus pernio, cutaneous granuloma, erythema nodosum, and subcutaneous nodules) occurs in 30% of patients (2, 3). Liver and spleen lesions are found in 5–15% of patients undergoing computed tomography (4, 5). Cardiac, bone, and neurological involvement are also possible but less frequent. However, cardiac involvement can be life-threatening and is the second cause of death from sarcoidosis after pulmonary involvement (1). Neurosarcoidosis is also an important cause of morbidity and mortality, especially in young patients (6–9), and occurs in 5–20% of patients (10). Neurosarcoidosis may affect cranial/peripheral nerves, brain, leptomeninges, spinal cord, and muscles (10–12). Clinical presentations are various; including facial nerve palsy, optic neuritis, aseptic meningitis, and lesions of the central nervous system inducing focal neurological deficits, hydrocephalus, encephalopathy, psychosis, peripheral neuropathy, and myopathy (9–12). Neurosarcoidosis is often associated with systemic sarcoidosis but isolated neurosarcoidosis is also described (6–10).

The diagnosis of sarcoidosis and especially neurosarcoidosis is challenging. There are many alternative causes of granulomatosis such as infection (e.g., mycobacterium tuberculosis), inflammatory diseases (e.g., inflammatory bowel diseases, granulomatosis with polyangiitis), and lymphoma (e.g., Hodgkin's lymphoma) which must be ruled out (9). A comprehensive diagnostic workup is necessary and a tissue biopsy is often required to confirm the diagnosis (9, 13). The diagnostic criteria of sarcoidosis, which have been recently updated (14), are based on the combination of a compatible clinical presentation, the presence of non-necrotizing granulomatous inflammation, and the exclusion of other causes of granulomatous diseases. Recently, the Neurosarcoidosis Consortium Consensus Group (NCCC) proposed new diagnostic criteria, to optimize the diagnosis of neurosarcoidosis and to enhance the clinical care of patients with suspected neurosarcoidosis (13). According to these criteria, the diagnosis of neurosarcoidosis is classified as follows: (i) possible when there are compatible clinical and radiological features without pathologic confirmation, (ii) probable when there is a pathologic confirmation of systemic granulomatous disease, and (iii) definite when there is a nervous system biopsy consistent with neurosarcoidosis (with or without systemic sarcoidosis) (13).

Treatment guidelines for neurosarcoidosis are mainly based on small cohort studies and non-randomized clinical trials, as there is a lack of robust randomized clinical trials. First-line treatment consists of corticosteroid therapy followed by methotrexate, azathioprine, or mycophenolate mofetil as corticosteroid-sparing second-line therapy. Cyclophosphamide has been used in the past for treating refractory sarcoidosis but is nowadays less considered due to its potential heavy side effects (bone marrow suppression, infection, infertility, hemorrhagic cystitis, and malignancy). Cyclosporine A has also been used but should not be preferred due to its safety profile (high blood pressure, renal impairment, and tremor) (15–17).

Based on the role of tumor necrosis factor-alpha (TNF-α) in autoimmune disease, anti-TNF-α monoclonal antibodies have been used as a novel therapeutic approach and are associated with favorable results in many diseases such as rheumatoid arthritis, psoriatic arthritis, inflammatory bowel disease, and non-infectious uveitis (18), as well as in systemic sarcoidosis (19–23). Recent studies provided class IV evidence that TNF-α antagonists are also beneficial in neurosarcoidosis (16, 24–26). They are currently proposed as third-line therapy in the management of aggressive and/or refractory neurosarcoidosis (10, 15, 27–38).

The objectives of our study are to describe the clinical and paraclinical features of neurosarcoidosis patients followed in a single Belgian academic center and to perform a comparative literature review of neurosarcoidosis, with a focus on treatment outcomes, in particular with the use of TNF-α antagonists.

## Materials and methods

### Patient selection and inclusion criteria

The study was conducted at Cliniques Universitaires Saint-Luc, UCLouvain (Belgium). All files of adult sarcoidosis followed in the departments of Internal Medicine and Neurology until March 2022 were retrospectively reviewed. Only patients diagnosed with possible, probable, or definite neurosarcoidosis (both central and peripheral neurosarcoidosis) according to the Neurosarcoidosis Consortium Consensus Group's 2018 Diagnostic Criteria (13) were included for final analysis.

### Data collection

Data were extracted from each patient's clinical records and reviewed by HY and PS to confirm the diagnosis of neurosarcoidosis, according to Neurosarcoidosis Consortium Consensus Group's criteria. Data on baseline characteristics, demographic features, clinical manifestations, history of systemic and neurologic sarcoidosis, biological (serum and cerebrospinal fluid), radiological (spinal cord MRI, brain MRI, [18F]FDG-PET/CT, thoraco-abdominal CT scan, chest x-ray), histological and electromyography results, treatment regimens, disease course and outcome were systematically collected for all patients.

The baseline was defined as the date of neurosarcoidosis diagnosis. Biopsy-confirmed sarcoidosis was defined by the presence of non-caseating granulomas (13, 24). Duration of follow-up was defined as the time between neurosarcoidosis diagnosis and the most recent clinical assessment. Therapies were classified as first-, second-, and third-lines. First-line therapy consists of corticosteroid treatment, second-line therapy consists of immunosuppressive therapy with methotrexate, azathioprine, mycophenolate mofetil, cyclosporine A, or (hydroxy)-chloroquine, and third-line therapy either consists of cyclophosphamide or monoclonal antibodies (TNF-α inhibitors or B-cell targeted therapy) (10, 24).

Based on clinical and/or radiological features, treatment response and outcomes were classified as ≪ complete remission ≫, ≪ partial remission ≫, ≪ clinically and/or radiologically active disease≫, ≪ progressive disease ≫, ≪ relapse ≫ or ≪ mortality ≫. Favorable outcomes include complete and partial remission which were defined, respectively, by the absence or conversely the presence of residual symptoms, without the need for alternative immunosuppressive therapy (10). Relapse or progression was defined as clinical and/or radiological worsening, either subacute or chronic, due to either neurological or systemic manifestations of sarcoidosis requiring a therapeutic modification (25). Relapse was defined as reoccurrence during a stable phase or appearance of a new localization, while progression as slow worsening of residual symptoms (4).

### Ethical consideration

This study was approved by the local Ethics Committee of the Cliniques Universitaires Saint-Luc (Brussels, CEHF 2021/29OCT/452-SARCO2). No written consent form was required given the retrospective nature of the study.

### Statistical analysis

Quantitative variables were reported as median values with ranges while qualitative values were shown as numbers and percentages. Treatment responses were compared using Fisher's exact test for categorical variables.

### Literature review

**Figure 1:**
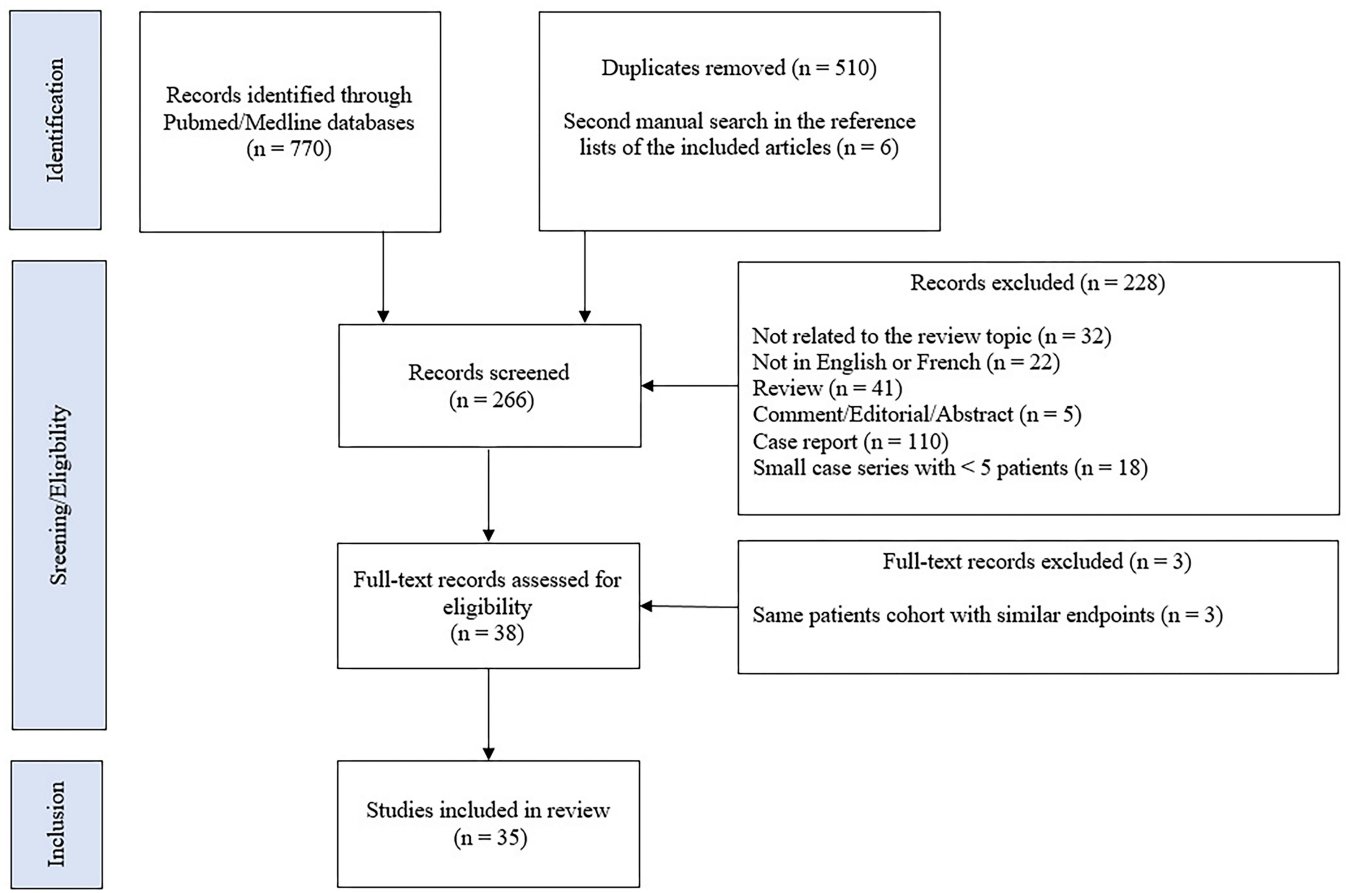
Flow diagram of included studies.

A comprehensive literature search was manually performed by searching the Pubmed/MEDLINE databases until 20 April 2022. We used the following terms: neurosarcoidosis OR (nervous system AND (sarcoidosis OR granulomatous disease OR sarcoid granuloma) AND (tumor necrosis factor OR TNF-alpha OR infliximab OR adalimumab OR certolizumab OR golimumab OR etanercept OR azathioprine OR methotrexate OR mycophenolate mofetil OR chloroquine OR cyclosporine OR cyclophosphamide OR rituximab OR thalidomide OR chlorambucil). Studies written in English or French were considered for inclusion, without date range restrictions. Original research articles were included if they reported at least five cases of possible, probable, or definite neurosarcoidosis, treated with second-line or third-line therapies. Studies were excluded if they reported pediatric cases or patients only treated with first-line therapy consisting of corticosteroid treatment. We conducted a second and manual search in the reference lists of the included articles. The title and abstract of the studies were independently screened by two reviewers (HY and PS) to ensure eligibility for inclusion. The flow diagram of included studies is shown in Figure 1. A pooled analysis of all available data was performed. The results are presented as the number for which the data are present out of the total number of patients for which the data were described [n/N *(%)*].

## Results

### Baseline characteristics

Among the 180 adult sarcoidosis patients followed in our tertiary center, 25 were identified as having neurosarcoidosis and 22 were included in the final analysis. Three patients were excluded: one patient had ocular and lymph node sarcoidosis with myopathy explained by concomitant myasthenia confirmed by neuromuscular biopsy, one had a diffuse glioneuronal tumor on brain biopsy, and one had altered consciousness explained by hypercalcemia without evidence of neurosarcoidosis. Patient characteristics are presented in Table 1 and Supplementary Table 1. Fourteen (64%) were male. The median age at the time of neurosarcoidosis diagnosis was 40.5 years (range 22–67) and the median time from onset of symptoms to diagnosis was 4 months (range 1–23). Except for one patient who met possible diagnostic criteria, all patients had histologically proven sarcoidosis from lymph nodes (*n* = 16), salivary glands (*n* = 1), spleen (*n* = 1), liver (*n* = 1), pituitary glands (*n* = 2), or brain parenchyma (*n* = 3) biopsy. Five (23%) patients were classified as having definite neurosarcoidosis and 16 (73%) with probable neurosarcoidosis. The median duration of follow-up was 3.6 years (range 0.2–17.4).

**Table 1:** Baseline characteristics of our patient cohort diagnosed with neurosarcoidosis.

**Cases**	**Sex, age (years)**	**Ethnicity**	**History of sarcoidosis**	**Systemic involvement**	**Neurological involvement**	**Neuro-sarcoidosis**	**Abnormal Brain MRI**	**Abnormal Spinal cord MRI**	**Abnormal FDG-PET/CT**	**Biopsy site**	**Biopsy results**
1	M, 53	C	No	LN, E, J, S	M, P, NE, SC, PN	Probable	Yes	Yes	Yes	Spleen	+
2	M, 27	N-A	No	LN	NE	Definite	Yes	NA	Yes	Pituitary gland	+
3	M, 67	A	No	L, LN, J	M, P, NE	Probable	Yes	No	Yes	LN	+
4	M, 40	C	No	No	CN, P	Definite	Yes	No	No	Brain parenchyma	+
5	M, 38	N-A	No	L, LN, J	M	Probable	No	NA	Yes	LN	+
6	M, 35	N-A	No	LN	SC	Probable	NA	Yes	Yes	LN	+
7	M, 55	A	No	L, LN, E, SG	CN, M, P	Possible	Yes	NA	NA	LN/Salivary glands	–/–
8	F, 22	A	No	LN, E	CN, M	Probable	Yes	NA	Yes	LN	+
9	M, 42	C	Yes	L, LN, C, B, H, S	NE	Probable	Yes	NA	Yes	LN/Liver	+/+
10	F, 26	C	No	No	NE	Definite	Yes	NA	No*	LN/Pituitary gland	Lymphoma/+
11	M, 32	C	No	No	M, P, NE	Definite	Yes	NA	No	Brain parenchyma	+
12	M, 41	C	No	LN	SC, PN, V	Probable	Yes	Yes	Yes	LN	+
13	M, 38	C	No	LN	M, P, SC	Probable	Yes	Yes	NA	LN	+
14	M, 49	C	Yes	L, LN, J	M, SC, My	Probable	No	Yes	Yes	LN	+
15	F, 40	C	Yes	L, LN, E, SG	CN, M, P	Probable	No	No	Yes	LN/Salivary glands	+/–
16	F, 39	N-A	No	LN, S	CN, P, V	Probable	Yes	NA	Yes	LN	+
17	F, 43	C	No	L, LN	M, P	Definite	Yes	No	Yes	LN/Brain parenchyma	+/+
18	F, 59	C	No	L, LN, C, E, J, S	CN, M, PN, My	Probable	Yes	NA	Yes	LN	+
19	F, 47	C	No	LN, J	M, P, SC	Probable	Yes	Yes	Yes	LN	+
20	F, 58	C	No	LN, E, J	CN, M, NE, SC	Probable	Yes	Yes	Yes	LN	+
21	M, 42	A	No	LN	PN, My	Probable	No	No	Yes	LN/Salivary gland/Skin	+/–/–
22	M, 25	C	No	LN, SG, H	CN, M, PN	Probable	No	No	Yes	LN/Salivary glands	+/+

NA, Not available; F, Female; M, Male; C, Caucasian; A, African; N-A, North-African; MRI, magnetic resonance imaging; EMG, Eletromyography; CSF, Cerebrospinal fluid; +, biopsy consistent with sarcoidosis; −, biopsy inconsistent with sarcoidosis; ACE, angiotensin-converting enzyme; L, Lungs; LN, Lymph nodes; C, Cutaneous; E, Eye; SG, Salivary glands; J, Joints; B, Bones; H, Hepatic; S, Spleen; H, Heart; CN, Cranial neuropathy; M, Meningeal involvement; P, Parenchymal disease; Hy, Hydrocephalus; NE, Neuro-endocrine; V, Vascular disease; SP, Spinal cord disease; PN, Peripheral neuropathy, My, Myopathy.

*Axillary and mediastinal lymph nodes attributed to lymphoma (confirmed by biopsy).

### Clinical characteristics

**Figure 2:**
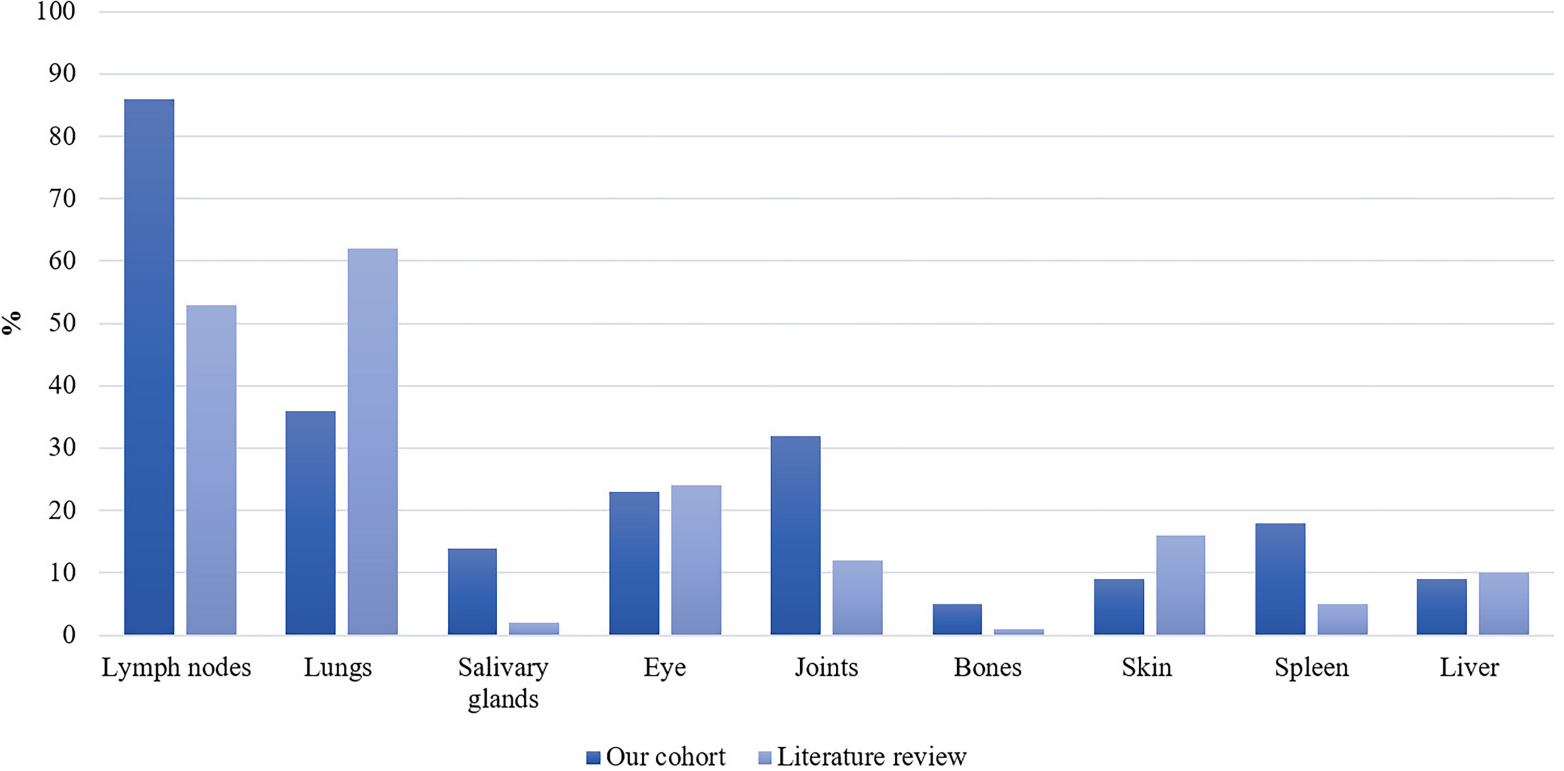
Proportion of systemic sarcoidosis involvement in our patient cohort (*n* = 22) and the literature (n/N = 907/1,793) expressed as percentages.

**Figure 3:**
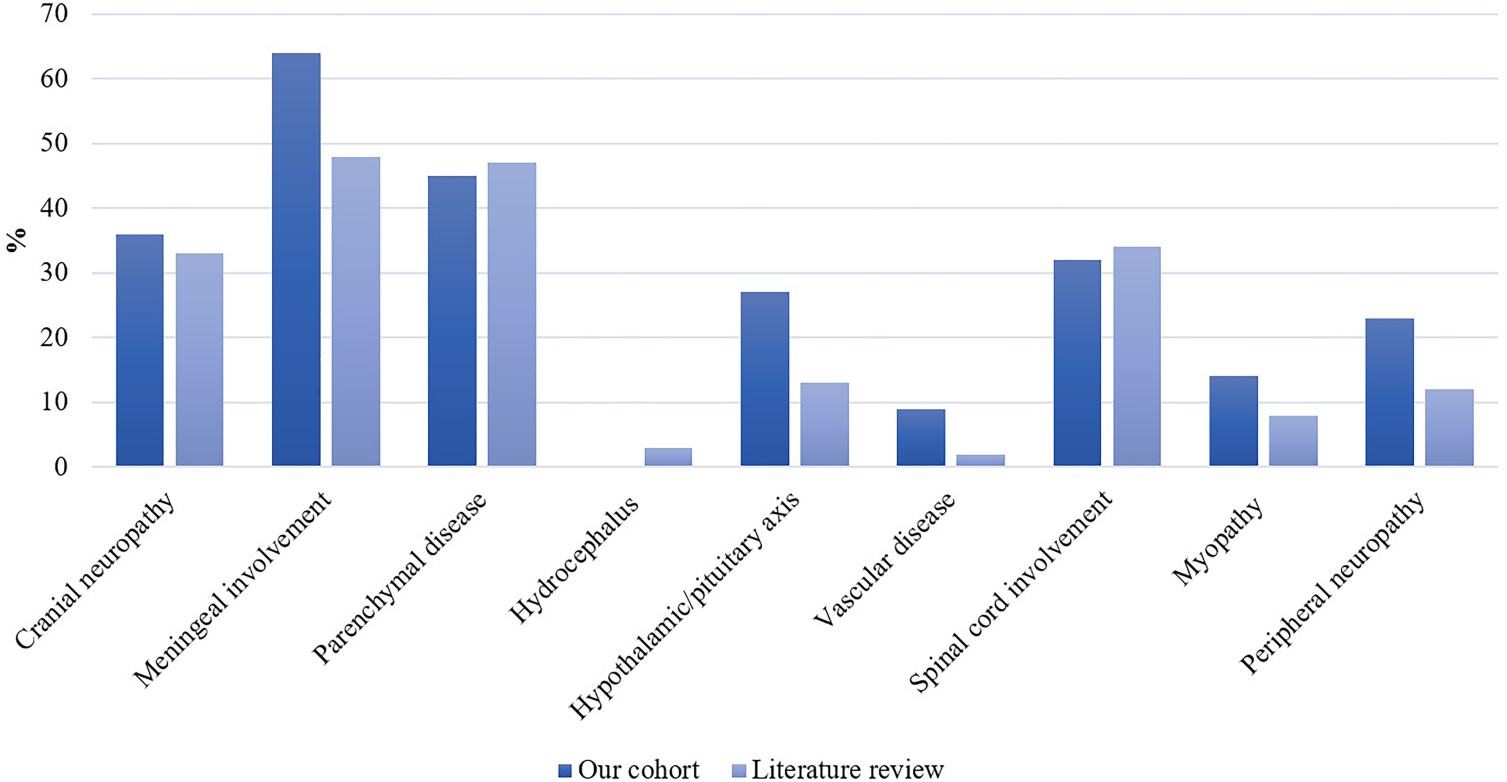
Distribution of neurological site involvement of neurosarcoidosis in our patient cohort (*n* = 22) and the literature as expressed as percentages. In the literature: n/N = 498/1,518 for cranial neuropathy; n/N = 722/1,507 for meningeal involvement; n/N = 622/1,334 for parenchymal disease; n/N = 40/1,237 for hydrocephalus; n/N = 162/1,237 for hypothalamic/pituitary axis; n/N = 24/1,237 for vascular disease; n/N = 426/1,237 for spinal cord involvement; n/N = 94/1,237 for myopathy; and n/N = 159/1,353 for peripheral neuropathy.

Clinical characteristics are reported in Table 2. Nineteen patients (86%) were diagnosed with systemic sarcoidosis, either before (*n* = 3) or concomitantly (*n* = 16) to the neurological involvement. Systemic sarcoidosis mainly consisted of lymph node, lung, and articular involvement; followed by ocular, splenic, salivary gland, skin, hepatic, and bone involvement (Figure 2). Ten (45%) patients had involvement of at least three systemic organs, while 5 (23%) had only lymph node involvement. Systemic symptoms mostly consisted of fatigue (36%) and arthralgia (36%), followed by weight loss, visual symptoms (diplopia, blurred vision), dyspnea, cough, and fever. Presenting neurological symptoms varied largely and consisted of a majority of headache (41%) and gait abnormalities (41%); followed by sensory abnormalities, including hypoesthesia, paresthesia, and neuropathic pain; and micturition abnormalities. The most commonly affected neurological site consisted of meningeal involvement (64%), including aseptic meningitis, leptomeningitis, and pachymeningitis, as well as parenchymal disease (45%), cranial nerve neuropathy (36%), and spinal cord involvement (32%) (Figure 3). Other neurosarcoidosis sites included the hypothalamic-pituitary axis, peripheral neuropathy, myopathy, and vascular disease, including ischemic and hemorrhagic stroke. Seventeen (77%) patients had multiple neurological involvement sites. Hypothalamic-pituitary axis involvement and aseptic meningitis were the unique manifestation in one and three patients, respectively, whereas isolated cranial neuropathy and hydrocephalus were not observed.

**Table 2:** Baseline and clinical characteristics of patients from our cohort and the literature.

		**Our cohort**	**Literature review**
Number of cases		22	1,793
Age at neurosarcoidosis diagnosis (years), median (range)		40.5 (22–67)	41.5 (26–70)*
Sex			
	Male, n/N (%)	14 (64)	777/1,682 (46)
	Female, n/N (%)	8 (36)	905/1,682 (54)
Ethnicity			
	Caucasian, n/N (%)	14 (64)	802/1,239 (65)
	African/North-African, n/N (%)	8 (36)	264/1,239 (21)
	Other, n/N (%)	0 (0)	135/1,239 (11)
	Unknown, n/N (%)	0 (0)	38/1,239 (3)
Neurosarcoidosis classification			
	Possible, n/N (%)	1 (5)	187/1,385 (13)
	Probable, n/N (%)	16 (73)	853/1,291 (66)
	Definite, n/N (%)	5 (23)	354/1,622 (23)
Isolated neurosarcoidosis, n/N (%)		3 (14)	220/1,331 (17)
Systemic sarcoidosis, n/N (%)		19 (86)	1,111/1,331 (83)
	History of systemic sarcoidosis, n/N (%)	3 (16)	214/637 (34)
	Systemic sarcoidosis at baseline, n/N (%)	16 (84)	179/578 (31)
	Primary neurological presentation, n/N (%)	0 (0)	220/1,331 (17)
Site of systemic involvement			
	Lymph nodes, n/N (%)	19 (86)	485/907 (53)
	Lungs, n/N (%)	8 (36)	568/907 (62)
	Ear-Nose-Throat, n/N (%)	0 (0)	69/907 (8)
	Salivary glands, n/N (%)	3 (14)	14/907 (2)
	Eye, n/N (%)	6 (27)	216/907 (24)
	Heart, n/N (%)	0 (0)	113/907 (12)
	Joints, n/N (%)	7 (32)	105/907 (12)
	Bones, n/N (%)	1 (5)	12/907 (1)
	Skin, n/N (%)	2 (9)	147/907 (16)
	Spleen, n/N (%)	4 (18)	47/907 (5)
	Liver, n/N (%)	2 (9)	89/907 (10)
	Kidney, n/N (%)	0 (0)	18/907 (2)
	Digestive tract, n/N (%)	0 (0)	12/907 (1)
	Scrotal, n/N (%)	0 (0)	4/907 (0.4)
Site of neurological involvement			
	Cranial neuropathy, n/N (%)	8 (36)	498/1,518 (33)
	Meningeal involvement, n/N (%)	14 (64)	722/1,507 (48)
	Parenchymal disease, n/N (%)	10 (45)	622/1334 (47)
	Hydrocephalus, n/N (%)	0 (0)	40/1,237 (3)
	Hypothalamic/pituitary axis, n/N (%)	6 (27)	162/1,237 (13)
	Vascular disease, n/N (%)	2 (9)	24/1,237 (2)
	Myelopathy/spinal cord involvement, n/N (%)	7 (32)	426/1,237 (34)
	Myopathy, n/N (%)	3 (14)	94/1237 (8)
	Peripheral neuropathy, n/N (%)	5 (23)	159/1,352 (12)

n/N represents the number for which the data are present out of the total number of patients for which the data were described.

^*^Available data (n/N = 1,545/1,793).

### Biological and radiological characteristics

Results of ancillary investigations at diagnosis are summarized in Table 3. Serum CRP and calcemia were elevated in 33 and 18% of patients, respectively. Serum lysozyme was elevated in 79% of patients compared to 16% with increased serum angiotensin-converting enzyme (ACE) levels. Lumbar puncture and CSF fluid analysis were performed in 15/22 patients and were abnormal in all patients. Pleocytosis (cell count > 5/mm^3^) was found in 80%, increased proteinorrhachia (>40 mg/dl) indicating blood-brain barrier dysfunction in 71%, low glucose levels in 9%, and CSF-specific IgG oligoclonal bands in 38% of patients. Brain MRI showed abnormalities in 16/21 (76%) patients mainly consisting of parenchymal lesions (63%), hypothalamic-pituitary axis lesions (38%), and meningeal enhancement (31%). Lesions were localized in the temporal lobe in 25% of patients. Spinal cord MRI showed abnormalities in 7/13 (54%) patients, revealing either longitudinally extensive myelitis or multiple disseminated spinal cord lesions. Lesions were predominantly located in the thoracic (86%), followed by the cervical (57%) and lumbar (29%) spine. [18F]FDG-PET/CT revealed systemic or neuro-sarcoidosis in 17/20 (85%) patients. Forty-one percent of these patients had a previously negative chest X-ray or thoracic CT scan.

**Table 3:** Paraclinical features of patients from our cohort and the literature at the time of neurological disease onset.

		**Our cohort**	**Literature review**
Serum analysis			
	CRP (mg/dl), median (range)	3.85 (0.1–71)	NA
	CRP increased, n/N (%)	7/21 (33)	7/21 (33)
	ACE (UECA), median (range)	51 (26–75)	73 (12–293)
	ACE increased, n/N (%)	3/19 (16)	311/839 (37)
	Lysozyme (mg/l), median (range)	23.9 (8.4–66)	35 (28–48)
	Lysozyme increased, n/N (%)	11/14 (79)	12/26 (46)
	Calcium (mmol/l), median (range)	2.45 (2.13–2.71)	2.39 (2.31–2.47)
	Calcium increased, n/N (%)	4/22 (18)	17/299 (59)
	Abnormal protein electrophoresis, n/N (%)	2/13 (15)	NA
Cerebrospinal fluid analysis			
	Lumbar puncture performed, n/N (%)	15/22 (68)	–
	White cell count (cells/mm3), median (range)	15 (2–33)	40 (0–648)
	Pleiocytosis, n/N (%)	12/15 (80)	528/832 (63)
	Protein (mg/dl), median (range)	58 (26–1,186)	105 (41–980)
	Proteinorachy, n/N (%)	10/14 (71)	563/807 (70)
	Hypoglycorachy, n/N (%)	1/11 (9)	123/371 (33)
	Increased IgG index, n/N (%)	1/8 (13)	18/49 (37)
	Oligoclonal bands present, n/N (%)	5/13 (38)	78/339 (23)
	Normal, n/N (%)	0/15 (0)	14/143 (10)
Abnormal imaging investigation			
	Chest X-ray, n/N (%)	2/9 (22)	130/267 (49)
	Thoracic CT scan, n/N (%)	6/7 (86)	58/118 (49)
	Abdnominal CT scan, n/N (%)	3/5 (60)	NA
	Brain CT scan, n/N (%)	0/1 (0)	20/37 (54)
	Brain MRI, n/N (%)	16/21 (76)	570/752 (76)
	Spinal MRI, n/N (%)	7/13 (54)	326/538 (61)
	[F18]FDG-PET CT, n/N (%)	17/20 (95)	137/319 (43)
	EMG, n/N (%)	4/10 (40)*	NA
Detailed abnormal brain MRI			
	Parenchymal lesions, n (%)	10/16 (63)	135/291 (46)
	Meningeal enhancement, n (%)	5/16 (31)	102/264 (39)
	Mass lesion, n (%)	2/16 (13)	7/82 (9)
	Cranial nerve enhancement, n (%)	3/16 (19)	21/151 (14)
	Hypothalamus/pituiary axis lesions, n (%)	6/16 (38)	39/169 (23)
	Vascular lesions, n (%)	2/16 (13)	NA
	Parietal location, n (%)	1/16 (6)	NA
	Temporal location, n (%)	4/16 (25)	NA
	Gadolinium enhancement, n (%)	8/16 (50)	69/132 (52)
Detailed abnormal spinal cord MRI			
	Longitudinally extensive myelitis, n (%)	3/7 (43)	66/137 (48)
	Multiple separated spinal cord lesions, n (%)	2/7 (29)	14/60 (23)
	Lesion location: cervical spine, n (%)	4/7 (57)	43/84 (51)
	Lesion location: thoracic spine, n (%)	6/7 (86)	38/67 (57)
	Lesion location: lumbar spine, n (%)	2/7 (29)	5/27 (19)
	Gadolinium enhancement, n (%)	7/7 (100)	101/140 (72)

n/N represents the number for which the data are present out of the total number of patients for which the data are described. NA, Not available; ACE, Angiotensin-converting enzyme; MRI, magnetic resonance imaging; EMG, electromyography.

*Abnormal EMG due to concomitant myasthenia (n = 1).

### Treatment

**Figure 4:**
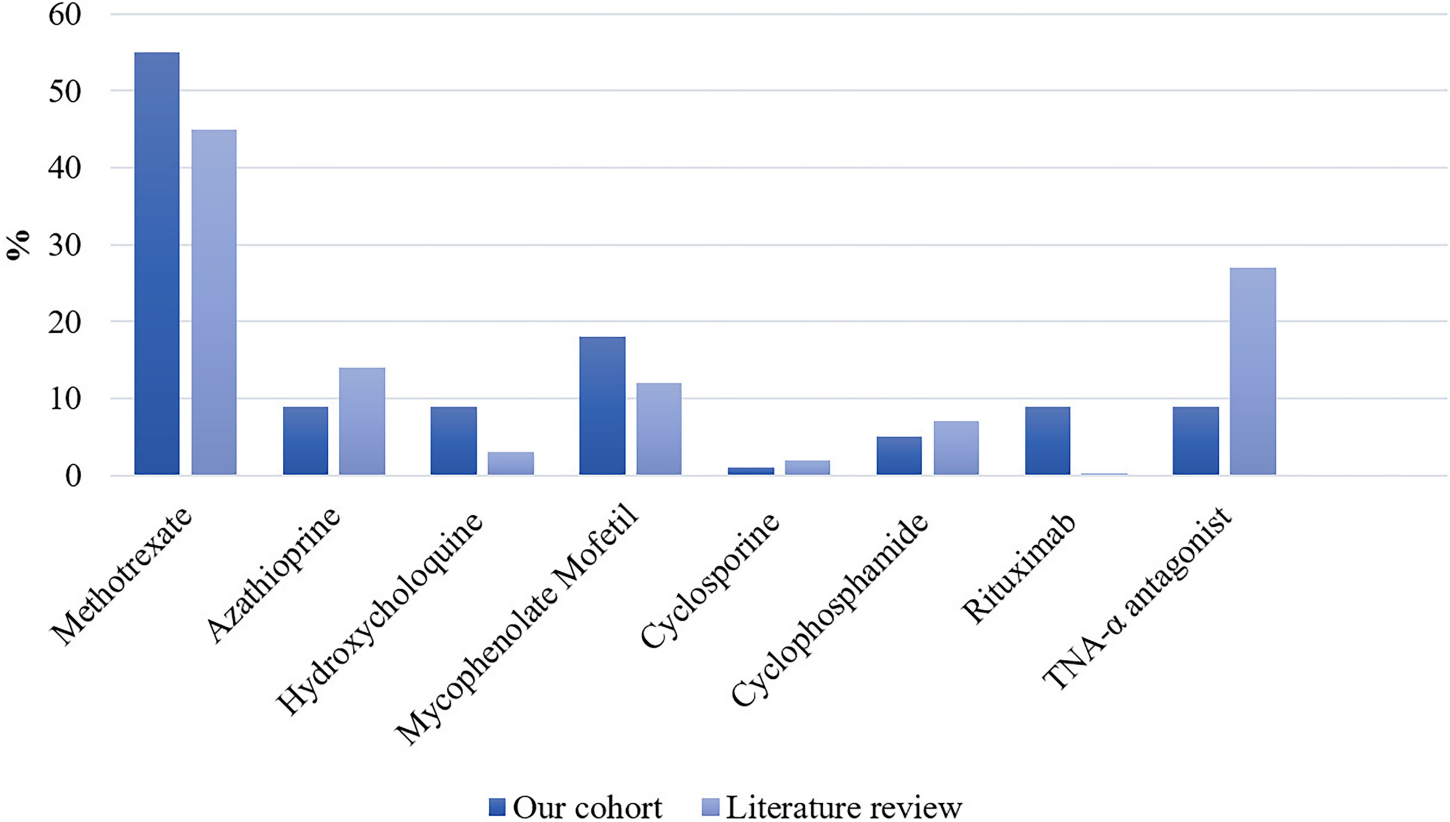
Proportion of second- and third-line therapies in our patient cohort (*n* = 22) and the literature expressed as percentages. In the literature: n/N = 557/1,248 for methotrexate; n/N = 185/1,363 for azathioprine; n/N = 44/1,431 for hydroxychloroquine; n/N = 178/1,431 for mycophenolate mofetil; n/N = 20/1,425 for cyclosporine; n/N = 99/1,431 for cyclophosphamide; n/N = 5/1,431 for Rituximab; and n/N = 405/1,494 for TNF-α antagonist.

**Figure 5:**
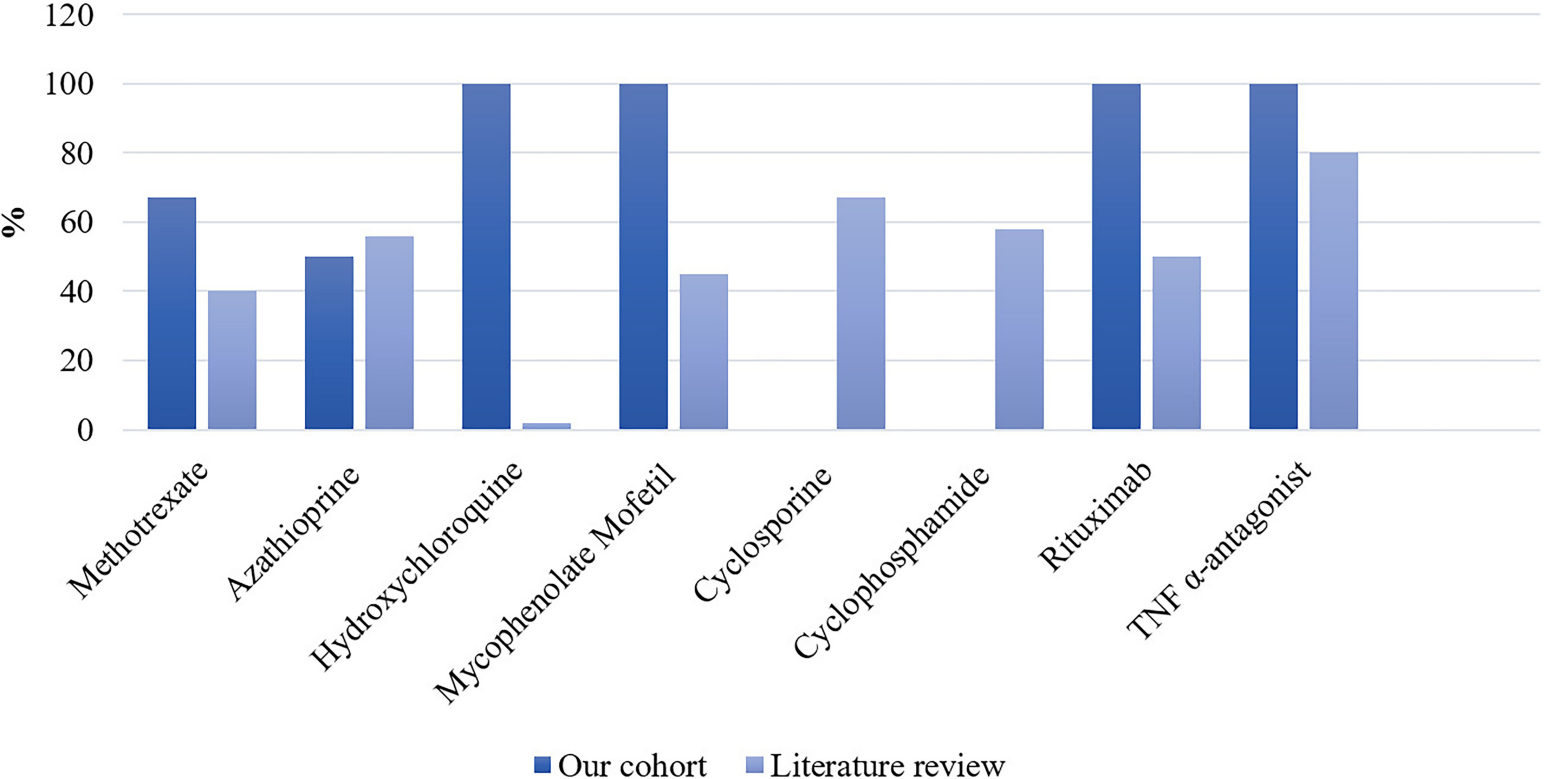
Proportion of favorable outcomes in patients from our cohort (*n* = 22) and the literature expressed as percentages. In the literature: n/N = 56/140 for methotrexate; n/N = 31/56 for azathioprine; n/N = 1/60 for hydroxychloroquine; n/N = 27/60 for mycophenolate mofetil; n/N = 10/15 for cyclosporine; n/N = 19/33 for cyclophosphamide; n/N = 2/4 for rituximab; and n/N = 280/352 for TNF-α antagonist.

Detailed treatments of patients are reported in Table 4. Initial therapy consisted of corticosteroids in all except in two patients; one was treated with methotrexate alone and one with ABVD (Doxorubicin, Bleomycin, Vinblastine, and Dacarbazine) for concomitant mediastinal lymphoma. Sixteen (73%) patients received second-line and 5 (23%) required intensification of treatment to third-line therapies (Table 5). Second-line therapy consisted of methotrexate in more than 50% of patients, followed by mycophenolate mofetil (18%), azathioprine (9%), and hydroxychloroquine (9%) (Figure 4). Methotrexate had a favorable outcome in 67% and azathioprine in 50% of patients, while hydroxychloroquine and mycophenolate mofetil in 100% of cases (Figure 5). A TNF-α antagonist was administered in 2 (9%) patients with 100% favorable outcomes. It was discontinued in 1/2 of patient because of remission and no relapse occurred following TNF-α antagonist discontinuation. Other treatment modalities consisted of hormonal substitution, anti-epileptic medication, and cervical decompression neurosurgery. The median cumulative dose of corticosteroids was 10.4 g, ranging from 2.9 to 33 g. At last follow-up, 75% of patients were corticosteroid-free. Eight patients (36%) experienced adverse events.

**Table 4:** Treatment and clinical outcomes of our patient cohort.

**Cases**	**Initial therapy**	**Maintenance immunosuppressive therapy**	**Cumulative corticosteroids dose (mg)**	**Relapse and/or deterioration during disease course**	**Adverse events**	**Other treatment modalities**	**Outcomes at last follow-up visite**	**Follow-up (years)**
1	Bolus CS	CYC, MMF	21,880	Yes	Yes	Hormonal substitution	Mortality**	1.4
2	Bolus CS	MTX	15,570	Yes	No	Hormonal substitution	Complete remission	3.1
3	Bolus CS	None	14,181	No	No	Hormonal substitution	Complete remission	4.2
4	Bolus CS	MTX, INF	21,109	Yes	Yes	Anti-epileptic medication	Partial remission	3.3
5	Bolus CS	HDQ, MTX	5,214	Yes	No	None	Complete remission	4.4
6	CS	None	NA	Yes	No	None	Partial remission	4.2
7	Bolus CS	None	10,314	No	Yes	None	Complete remission	17.4
8	CS	AZA, MMF, RTX	3,912	Yes	No	None	Complete remission	4
9	CS	None	3,922	No	No	None	Complete remission	4.3
10	ABVD*	None	NA	No	No	Hormonal substitution	Complete remission	3
11	CS	MTX	2,880	Yes	No	None	Partial remission	2.2
12	Bolus CS	MTX	33,050	Yes	Yes	Cervical decompression surgery	Progressive disease	6.3
13	Bolus CS	MTX	NA	No	No	None	Partial remission	8.6
14	Bolus CS	MTX, INF	11,952	Yes	Yes	None	Partial remission	7.8
15	CS	HDQ	9,768	Yes	No	None	Complete remission	6.1
16	CS	MTX	NA	Yes	Yes	None	Partial remission	2.4
17	MTX	MTX, RTX	NA	Yes	No	Pyridostigmin***	Partial remission	1.6
18	Bolus CS	MTX	10,415	Yes	Yes	None	Partial remission	1.3
19	CS	AZA, MMF	7,037	No	Yes	None	Partial remission	1
20	CS	MMF, MTX	18,079	Yes	Yes	None	Partial remission	3.8
21	CS	MTX	Lost to follow-up	Lost to follow-up	No	None	Lost to follow-up	0.2
22	CS	None	Lost to follow-up	Lost to follow-up	No	None	Lost to follow-up	0.3

NA, Not available; CS, Corticosteroids; MTX, Methotrexate; CYC, Cyclophosphamide; MMF, Mycophenolate Mofetil; INF, Infliximab; HCQ, Hydroxychloroquine; AZA, Azathioprine; RTX, Rituximab.

^*^ABVD (Doxorubicin, Bleomycin, Vinblastine, Dacarbazine) for concomitant lymphoma.

^**^Mortality unrelated to neurosarcoidosis.

^***^For concomitant myasthenia.

**Table 5:** Detailed treatment of our patient cohort.

No treatment, *n* (%)	0 (0)
First line therapy, *n* (%)	21 (95)
Second line therapy, *n* (%)	16 (73)
Third line therapy, *n* (%)	5 (23)
Detailed treatment
Corticosteroids, *n* (%)	21 (95)
Methotrexate, *n* (%)	12 (55%)
Azathioprine, *n* (%)	2 (9)
Hydroxychloroquine, *n* (%)	2 (9)
Mycophenolate Mofetil, *n* (%)	4 (18)**
Ciclosporine, *n* (%)	0 (0)
Cyclophosphamide, *n* (%)	1 (5)
Rituximab, *n* (%)	2 (9)
TNA-α antagonist, *n* (%)	2 (9)*
Treatment switches	
First to second or third line, *n* (%)	16 (73)
Second to third line, *n* (%)	4 (18)
Third to second line, *n* (%)	1 (5)
Between second line, *n* (%)	4 (18)
Between third line, *n* (%)	0 (0)
Other treatment modalidites, *n* (%)	6 (27)
Hormonal substitution, *n* (%)	4 (18)
Anti-epileptic medication, *n* (%)	1 (5)
Neurochirurgical intervention, *n* (%)	1 (5)
Corticosteroids-free at last-follow-up, *n* (%)	15 (75)
Corticosteroids cumulative dose (g), *n* (%)	10.4 (2.9–33)
Adverse events, *n* (%)	8 (36)
Follow-up (years), median (range)	3.6 (0.2–17.4)

^*^Infliximab (*n* = 2).

^**^One patient was treated twice with mycophenolate mofetil.

### Outcome

Two patients were lost to follow-up, and thus were not included in the outcome analysis. Seventy percent of patients experienced at least one relapse and/or progression during their disease course. At the last follow-up, 18 of 20 (82%) patients achieved complete (*n* = 8) or partial (*n* = 10) remission while one patient experienced progressive disease (Table 4). In patients with partial remission, residual symptoms were peripheral neuropathy, gait disorders, and cognitive impairment. One patient died during follow-up with a cause of death not related to neurosarcoidosis.

### Literature review

Our literature search identified 776 articles of which 741 were excluded after abstracts and full-text records screening (Figure 1). Thirty-five articles met our inclusion criteria, including 1,793 patients diagnosed with neurosarcoidosis from 1995 to 2021 (7, 16, 19–53). Only three (9%) studies were prospective (33, 36, 48) and 14 (40%) were multicentric (16, 19, 22, 24–26, 29, 30, 32, 35, 39, 40, 43, 45) (Supplementary Table 2). As illustrated in Table 2, patient characteristics are consistent with those in our cohort, except for male predominance. The Neurosarcoidosis Consortium Consensus Group's 2018 Diagnostic Criteria (13) were applied to all studies to consistently identify possible (13%), probable (66%), and definite (23%) neurosarcoidosis. Eighty-three percent of patients were diagnosed with systemic sarcoidosis, mainly consisting of lungs (62%) and lymph nodes (53%) involvement. In contrast to our patient cohort, cardiac, ear-nose-throat, kidney, digestive tract, and scrotal localizations were also described. Similar proportions in systemic and neurological symptoms as well as site of neurological involvement were observed in our cohort and in the literature review except for hydrocephalus described in 3% of patients in the literature (Figure 3). Results of ancillary investigations at diagnosis are summarized in Table 3. Serum lysozyme and ACE were elevated in 46 and 37% of patients, respectively. Pleocytosis was found in 63%, increased proteinorrhachia in 70%, low glucose levels in 33%, increased IgG index in 37%, and CSF-specific IgG oligoclonal bands in 23% of patients. Brain MRI showed abnormalities in 76% of patients mainly consisting of parenchymal lesions (46%), meningeal enhancement (39%), and hypothalamic-pituitary axis lesions (23%), similar to our cohort. Spinal cord MRI showed abnormalities in 61% of evaluated patients, while [18F]FDG-PET/CT revealed systemic or neuro-sarcoidosis in 43% of patients. Second-line therapy mainly consisted of methotrexate (45%), followed by azathioprine (14%) and mycophenolate mofetil (12%) (Figure 4). In most cases, these therapies were initiated for treating a progressive or relapsing disease as well as in association with a TNF-α antagonist (Table 6). A favorable outcome was reported in 40% of patients treated with methotrexate, in 56% with azathioprine, and in 45% with mycophenolate mofetil, as illustrated in Figure 5, Tables 6, 7. Relapse or progressive disease occurred most frequently with mycophenolate mofetil (58%) and azathioprine (46%) compared to methotrexate (39%). Third-line therapy consisted of TNF-α antagonists in 27% of patients with a high rate of favorable outcome (80%), similarly to our cohort. The proportion of favorable outcomes was significantly higher in patients treated by the TNF-α antagonist compared to those treated by methotrexate (*p* < 0.0001), mycophenolate mofetil (*p* < 0.0001), or azathioprine (*p* < 0.0001) (Table 8). The final outcome in the literature was reported in 1,446/1,793 patients, with favorable outcomes in 65% of cases.

**Table 6:** Indication, outcome, reasons for discontinuation, and adverse events according to the second-line therapy at baseline and during follow-up in patients from our cohort and the literature.

		**Second line therapy**							
		**Methotrexate**		**Azathioprine**		**Mycophenolate mofetil**		**Cyclosporine**	
		**Our cohort (*n* = 12)** ***n* (%)**	**Literature review (*n* = 557)** **n/N (%)**	**Our cohort** **(*n* = 2)** ***n* (%)**	**Literature review** **(*n* = 185)** **n/N (%)**	Our cohort (*n* = 5)* ***n* (%)**	**Literature review (*n* = 178)** **n/N (%)**	**Our cohort (*n* = 0)** ***n* (%)**	**Literature review (*n* = 20)** **n/N (%)**
Indication									
	Since baseline	2 (17)	16/356 (4)	0 (0)	8/143 (6)	1 (20)	6/90 (7)	0 (0)	0/16 (0)
	Active, progressive or relapsing disease	5 (42)	35/356 (10)	1 (50)	36/143 (25)	3 (60)	18/90 (20)	0 (0)	16/16 (100)
	Corticosteroids sparing	3 (25)	20/356 (7)	1 (50)	8/143 (6)	0 (0)	13/90 (14)	0 (0)	0/16 (0)
	Intolerance to other treatment	1 (8)	12/356 (3)	0 (0)	6/143 (4)	1 (20)	6/90 (7)	0 (0)	0/16 (0)
	Associated with TNF-alpha antagonists	1 (8)	83/356 (23)	0 (0)	32/143 (22)	0 (0)	28/90 (31)	0 (0)	0/16 (0)
	Unknown	0 (0)	190/356 (53)	0 (0)	53/143 (37)	0 (0)	19/90 (21)	0 (0)	0/16 (0)
Outcomes									
	Favorable outcome	8 (67)	56/140 (40)	1 (50)	31/56 (56)	5 (100)	27/60 (45)	0 (0)	10/15 (67)
	Active, progressive or relapsing disease	4 (33)	45/140 (32)	1 (50)	13/56 (23)	0 (100)	25/60 (42)	0 (0)	3/15 (20)
	Mortality	0 (0)	0/140 (0)	0 (0)	3/56 (5)	0 (100)	0/60 (0)	0 (0)	1/15 (7)
	Unknown	0 (0)	39/140 (28)	0 (0)	9/56 (16)	0 (100)	8/60 (13)	0 (0)	0/15 (0)
Discontinuation		5/12 (42)	57/127 (45)	2 (100)	24/87 (26)	4 (80)	38/64 (59)	0 (0)	11/15 (73)
	Intolerance or adverse events	0/5 (0)	14/57 (25)	1 (50)	4/24 (17)	2 (50)	3/38 (8)	0 (0)	2/11 (18)
	Relapse or progressive disease	2/5 (40)	22/57 (39)	1 (50)	11/24 (46)	0 (0)	22/38 (58)	0 (0)	0/11 (0)
	Complete or partial remission	3/5 (60)	15/57 (26)	0 (0)	5/24 (21)	2 (0)	9/38 (24)	0 (0)	0/11 (0)
	Study protocol	0 (0)	0 (0)	0 (0)	0/24 (0)	0 (0)	0/38 (0)	0 (0)	6/11 (55)
	Unknown	0/5 (0)	6/57 (10)	0 (0)	4/24 (17)	0 (0)	4/38 (10)	0 (0)	3/11 (27)
Adverse events		0 (0)	21/246 (9)	1 (50)	5/62 (8)	1 (25)	1/18 (6)	0 (0)	5/15 (33)

n/N represents the number for which the data are present out of the total number of patients for which the data are described.

NA, Not available.

*One patient was treated two times with mycophenolate mofetil.

Table adapted from Gavoille et al. (25).

**Table 7:** Indication, outcome, reasons for discontinuation, and adverse events according to the third-line therapy at baseline and during follow-up in patients from our cohort and the literature.

	**Cyclophosphamide**		**Rituximab**		**TNF-alpha antagonist**	
	**Our cohort (*n* = 1)** ***n* (%)**	**Literature review (*n* = 99)** **n/N (%)**	**Our cohort (*n* = 2)** ***n* (%)**	**Literature review (*n* = 5)** **n/N (%)**	**Our cohort** **(*n* = 2)** ***n* (%)**	**Literature review (*n* = 404)** **n/N (%)**
Indication						
Since baseline	0 (0)	22/70 (31)	0 (0)	0/1 (0)	0 (0)	8/260 (3)
Active, progressive or relapsing disease	1 (100)	41/70 (59)	2 (100)	0/1 (0)	1 (50)	173/260 (66)
Corticosteroids sparing	0 (0)	0/70 (0)	0 (0)	1/1 (100)	0 (0)	15/260 (6)
Intolerance to other treatment	0 (0)	1/70 (1)	0 (0)	0/1 (0)	0 (0)	12/260 (5)
Associated with TNF-alpha antagonists	0 (0)	1/70 (1)	0 (0)	0/1 (0)	–	–
Unknown	0 (0)	5/70 (7)	0 (0)	0 (0)	1 (50)	52/260 (20)
Outcomes						
Favorable outcome	0 (0)	19/33 (58)	2 (100)	2/4 (50)	2 (100)	280/352 (80)
Active, progressive or relapsing disease	1 (100)	9/33 (27)	0 (0)	2/4 (50)	0 (0)	39/352 (11)
Mortality	0 (0)	0/33 (0)	0 (0)	0/4 (0)	0 (0)	1/352 (0,3)
Unknown	0 (0)	5/33 (15)	0 (0)	0/4 (0)	0 (0)	32/352 (9)
Discontinuation	1 (100)	12/58 (21)	1 (50)	NA	1 (50)	69/221 (31)
Intolerance or adverse events	0 (0)	3/12 (25)	0 (0)	–	0 (0)	11/69 (16)
Relapse or progressive disease	1 (100)	2/12 (17)	0 (0)	–	0 (0)	4/69 (6)
Complete or partial remission	0 (0)	0/12 (0)	1 (100)	–	1 (100)	48/69 (69)
Study protocol	0 (0)	0/12 (0)	0 (0)	–	0 (0)	0/69 (0)
Unknown	0 (0)	7/12 (58)	0 (0)	–	0 (0)	6/69 (9)
Adverse events	0 (0)	5/11 (45)	0 (0)	NA	1 (50)	59/204 (29)

n/N represents the number for which the data are present out of the total number of patients for which the data are described.

NA, Not available.

Table adapted from Gavoille et al. (25).

**Table 8:** Comparison of treatment outcomes of neurosarcoidosis according to treatment between azathioprine, methotrexate, and mycofenolate mofetil, respectively, and TNF-alpha antagonist.

	**Methotrexate**	**Anti-TNF alpha**	**Odds ratio**	**95% CI**	***p*-value**
Favorable outcome	56/140	280/352	4.68	3.10–7.03	<0.0001
	**Mycophenolate mofetil**	**Anti-TNF alpha**	**Odds ratio**	**95% CI**	***p*** **-value**
Favorable outcome	27/60	280/352	5.89	3.58–9.86	<0.0001
	**Azathioprine**	**Anti-TNF alpha**	**Odds ratio**	**95% CI**	***p*** **-value**
Favorable outcome	31/56	280/352	5.26	2.84–9.36	<0.0001

Proportions are indicated and were compared using Fisher's exact test. Reciprocal odds ratios for a favorable outcome on anti-TNF-a therapy are indicated, together with a 95% confidence interval and p-value.

Patients from the published cohort and ours are combined.

### Focus on neurosarcoidosis treatment with TNF-α antagonists

Including our study, we identified 25 studies reporting 406 patients diagnosed with neurosarcoidosis and treated with TNF-α antagonists (7, 16, 19–28, 36, 39, 41, 42, 44–53) (Supplementary Table 3). Detailed patient characteristics and treatment modalities are reported in Table 9. Ninety-seven percent of patients received intravenous infliximab at a dose of 5 mg/kg (ranging from 3.5 to 7 mg/kg) initially given at 2- or 4-week intervals followed by every 6 or 8 weeks. Three percent of patients received 40 mg of subcutaneous adalimumab every 1 or 2 weeks. Anti-TNF-α treatment indication mainly consisted of relapse or progression under other therapy (66%). Eighty-eight percent of patients were concomitantly treated with corticosteroids. Other accompanying treatments included methotrexate (28%), azathioprine (11%), and mycophenolate mofetil (10%) in most cases. Seven percent of patients had no concomitant treatment. The median treatment duration was 23 months (1–93) with a median follow-up of 29 months (1–123 months). Eighty percent presented favorable outcomes following anti-TNF-α therapy, while mortality related to neurosarcoidosis was reported in only one patient (24). Corticosteroids could be, respectively, tapered or stopped in 36% and 34% of patients. Two studies, including a total of 38 patients, reported a significant decrease in the daily dose of corticosteroids (*p* < 0.0001) (45, 46). Anti-TNF-α treatment was discontinued in 31% of patients because of stable disease (70%), intolerance or adverse events (16%), and relapse or progression (6%). Data post-treatment discontinuation was available in 28 patients. Among these, 50% of patients presented a relapse after stopping anti-TNF-α therapy. Twelve patients were rechallenged with either infliximab (*n* = 11) or adalimumab (*n* = 1) and all showed favorable outcomes. Seven studies reported switches from Infliximab to Adalimumab (*n* = 8), Adalimumab to Infliximab (*n* = 1), and Etanercept to Infliximab (*n* = 1). Indications were mainly adverse events (28%) and relapse or progression (33%). A favorable outcome was noted in 94% of these patients. Overall, adverse events were reported in 29% of patients, including infection, infusion reaction, and headache in most cases.

**Table 9:** Combined disease characteristics, management, and outcomes of neurosarcoidosis cases treated with the anti-TNF-α antagonist from our cohort (*n* = 2) and the literature (*n* = 404).

Cases, n	406
Sex	
Male, n/N (%)	90/183 (49%)
Female, n/N (%)	93/183 (51%)
Systemic sarcoidosis, n/N (%)	121/132 (92%)
Site of neurological involvement	
Meningeal involvement, n/N (%)	87/160 (54%)
Parenchymal disease, n/N (%)	45/160 (28%)
Cranial neuropathy, n/N (%)	38/160 (24%)
Spinal cord involvement, n/N (%)	69/160 (43%)
TNF-α antagonist treatment	
Infliximab, n/N (%)	368/379 (97%)
Adalimimumab, n/N (%)	10/379 (3%)
Both infliximab and adalimumab, n/N (%)	1/379 (0,3%)
TNF-α antagonist indication	
Relapse/progression under other therapy, n/N (%)	173/262 (66%)
Maintenance or corticosteroid-sparing therapy, n/N (%)	16/262 (6%)
Baseline therapy due to severe disease phenotype, n/N (%)	8/262 (3%)
Intolerance to other treatment, n/N (%)	13/262 (5%)
Unknown, n/N (%)	52/262 (20%)
Prior treatment	
Corticosteroids, n/N (%)	185/289 (64%)
Methotrexate, n/N (%)	78/289 (27%)
Azathioprine, n/N (%)	42/289 (15%)
Mycophenolate mofetil, n/N (%)	50/289 (17%)
Cyclophosphamide, n/N (%)	32/289 (11%)
Hydroxychloroquine, n/N (%)	6/289 (2%)
Cyclosporine, n/N (%)	2/289 (0.7%)
Rituximab, n/N (%)	1/289 (0.3%)
Etanercept, n/N (%)	1/289 (0.3%)
None, n/N (%)	8/289 (3%)
Concomitant treatment	
Corticosteroids, n/N (%)	242/275 (88%)
Methotrexate, n/N (%)	78/275 (28%)
Azathioprine, n/N (%)	30/275 (11%)
Mycophenolate mofetil, n/N (%)	28/275 (10%)
Hydroxychloroquine, n/N (%)	1/275 (0.3%)
Cyclophosphamide, n/N (%)	1/275 (0.3%)
None, n/N (%)	19/275 (7%)
Outcome	
Favorable outcome n/N (%)	282/354 (80)
Relapse or progression, n/N (%)	39/354 (11)
Mortality, n/N (%)	1/354 (0,3%)
Unknown, n/N (%)	32/354 (9%)
Corticosteroids tapering or stopping, n/N (%)	77/110 (70%)
Discontinuation of TNF-α antagonist, n/N (%)	70/223 (31%)
Intolerance or adverse events, n/N (%)	11/70 (16%)
Relapse or progression, n/N (%)	4/70 (6%)
Stable disease, n/N (%)	49/70 (70%)
Unknown, n/N (%)	6/70 (8%)
Relapse post-TNF-α antagonist discontinuation, n/N (%)	14/28 (50%)
Duration of treatment (months), median (range)	23 (1–93)
Adverse events, n/N (%)	60/206 (29%)
Follow-up (months), median (range)	29 (1–123)

n/N represents the number for which the data are present out of the total number of patients for which the data are described.

## Discussion

We retrospectively described clinical features, ancillary investigations, and treatment in a cohort of patients with neurosarcoidosis treated in a tertiary academic hospital in Belgium and compared our results with the existing evidence published so far in the literature, with a focus on treatment outcomes with TNF-α antagonists.

### Clinical characteristics

More than 80% of patients with neurosarcoidosis have associated systemic sarcoidosis, mainly consisting of lungs and lymph nodes involvement. Neurologic manifestations are the initial clinical symptoms in 50–70% of patients and systemic sarcoidosis is subsequently detected during the diagnostic workup. Our data confirm the large diversity and heterogeneity in the clinical presentation of neurosarcoidosis. Meninges are the most frequently affected neurological site and may be complicated by cranial nerve dysfunction and seizures as well as hydrocephalus in case of chronic meningitis (9). The involvement of brain parenchyma can explain acute or chronic cognitive dysfunction, headache, seizures, gait disturbances, stroke, and hydrocephalus (54). Cranial nerve neuropathy is also part of the commonly reported manifestation of neurosarcoidosis, either affecting the optic, the facial, or the vestibulocochlear nerves (9, 54). Spinal cord is involved in one-third of patients and may lead to motor or sensory deficits, bowel and bladder dysfunction, as well as sexual dysfunction (9, 54). Other neurosarcoidosis sites reported to be involved included the hypothalamic-pituitary axis resulting in hormonal deficiencies (e.g., syndrome of inappropriate antidiuretic hormone, hypothyroidism, hyperprolactinemia, hypoadrenalism, and diabetes insipidus) (9, 54), the peripheral nervous system, and muscles (9).

### Ancillary investigations

The diagnosis of neurosarcoidosis is challenging, largely due to heterogeneous clinical presentations and low sensitivity of ancillary investigations (10). The diagnostic criteria of neurosarcoidosis have been updated in 2018 and categorized patients into definite, probable, and possible neurosarcoidosis based on suggestive clinical presentation, results of ancillary investigations, histopathological confirmation of non-caseating granulomas, and rigorous exclusion of other causes (9, 10, 13).

In accordance with previous data (9, 10, 42, 53, 54), we confirm the low sensitivity of serum analysis except for lysozyme levels which were increased in almost 80% of our cohort, while it was reported abnormal in only half of patients with neurosarcoidosis in the literature (42). Serum testing is therefore mainly useful to exclude alternative diagnoses such as autoimmune and infectious diseases (tuberculosis, syphilis) and systemic complications in the context of sarcoidosis (liver and kidney impairment as well as hypercalcemia and hematological abnormalities). Therefore, the initial biological workup is classically characterized by: CRP, calcium, antinuclear antibody, antineutrophil cytoplasmic antibody, HIV, and syphilis serologies, as well as screening for tuberculosis. In some cases (history of traveling, immunosuppression, and contact with animals such as cat and sheep), fungal and bacterial serologies (bartonella and brucella) may be indicated according to clinical suspicion (9). Anti-aquaporine-4 and anti-myelin oligodendrocyte glycoprotein IgG antibodies should be measured especially in the context of myelitis. There remains an unmet need to define novel biomarkers to help in establishing the diagnosis of neurosarcoidosis. In 2019, serum soluble IL-2 receptor was proposed to be a sensitive diagnostic biomarker (55) but its cut-off levels have not been precisely defined yet (56).

Although unspecific in neurosarcoidosis (9, 10, 41, 53), lumbar puncture should be considered to evaluate intrathecal inflammation and to exclude alternative diagnoses (9). Many patients with neurosarcoidosis have an abnormal cerebrospinal fluid (CSF) analysis, including pleocytosis (mostly mild to moderate, with lymphocytic predominance), increased protein, and rarely low glucose levels (10, 57). As neurosarcoidosis is a rare non-infectious disease that can cause hypoglycorrhachia (9), it may have a diagnostic value after the exclusion of lymphoma mycobacterial and fungal infection (41, 57). It could be particularly relevant in sarcoidosis with spinal cord involvement as hypoglycorrhachia is not observed in other cases of inflammatory myelopathies (57). An elevated immunoglobulin G index and IgG oligoclonal bands are described in about one-third of patients with neurosarcoidosis but should be interpreted with caution as it occurs in 95% to 98% of patients with multiple sclerosis (50).

In the evaluation of neurosarcoidosis, brain and spinal MRI with gadolinium injection is the gold-standard imaging modality (9, 54, 57) due to its high sensitivity (82–97%) for active inflammation (54). The value of the [18F]FDG-PET/CT is particularly well-illustrated in our cohort as 85% of patients had abnormalities, although a significant proportion of these had a normal chest X-Ray or CT scan. Its usefulness is based on the detection of extra-neurologic localizations and the identification of hypermetabolic target lesions easily accessible for biopsy (9, 25, 58).

Neurosarcoidosis is fundamentally a diagnosis made by histopathology, although there is also a histological differential diagnosis to make (9). The definite diagnostic criteria of neurosarcoidosis are met in a minority of patients as it requires relatively high-risk invasive procedures such as brain or leptomeningeal biopsy (13). Diagnosis of probable neurosarcoidosis is therefore preferentially obtained by less invasive extraneural biopsy, such as pulmonary, lymph node, salivary gland, or skin biopsy (9, 54). Actually, there are many alternative causes of granulomatosis such as infection (e.g., mycobacterium tuberculosis), inflammatory diseases (e.g., inflammatory bowel diseases and granulomatosis with polyangiitis), and lymphoma (e.g., Hodgkin's lymphoma), which must be ruled out (9).

### Treatment

Treatment guidelines for neurosarcoidosis are principally based on expert opinion and observations from small cohort studies and non-randomized clinical trials (9, 10, 54). Treatment of neurosarcoidosis should therefore be patient-tailored and take into consideration other concomitant systemic involvement (9). Early and aggressive treatment is required in the majority of neurosarcoidosis cases to prevent morbidity and mortality (9, 38, 54), except in cases of isolated facial nerve palsy or aseptic meningitis, in which moderate and shorter treatment courses may be sufficient (27, 28, 38, 48).

Corticosteroids remain the cornerstone and first-line treatment in neurosarcoidosis (9, 28, 54, 59). However, due to incomplete response, disease progression, recurrence, or corticosteroid-induced toxicity, second- and/or third-line therapies are required in a majority of patients as was the case in our patient cohort and the literature review. Methotrexate is the most frequently used second-line treatment. Azathioprine, mycophenolate mofetil, and hydroxychloroquine are usual alternatives to methotrexate but are associated with lesser efficacy in relapse prevention (7, 25, 28, 43, 47). Cyclosporine and cyclophosphamide are less considered due to their significant side effects and should therefore be used as a last resort (16, 17, 28).

Third-line treatments such as TNF-α antagonists are increasingly used in the management of neurosarcoidosis. Infliximab and adalimumab are monoclonal antibodies that inactivate TNF-α, a pro-inflammatory cytokine critical for the formation and maintenance of sarcoid granulomas (9, 16, 27, 52). They are commonly used in combination with corticosteroids and other immunosuppressive therapy such as methotrexate and azathioprine, although their effectiveness as monotherapy in neurosarcoidosis is also reported (26). In addition to potential synergistic immunosuppressive benefits, the combination of a TNF-α antagonist with low-dose second-line therapy may be useful to attenuate the risk of anti-drug antibody formation (16, 26, 27, 36, 37). Most patients (80%) achieve a favorable outcome with anti-TNF-α therapy. The proportion of favorable outcomes was significantly higher in patients treated with TNF-α antagonist compared to those treated with methotrexate, mycophenolate mofetil, or azathioprine. In up to 70% of patients, corticosteroids could be tapered or even stopped, confirming the role of anti-TNF-α as efficient corticosteroid-sparing agents even in cases of refractory or aggressive neurosarcoidosis (9, 27). Although rare, relapses can occur during anti-TNF-α therapy. In this circumstance, it is important to verify the presence of anti-drug-neutralizing antibodies (24). When anti-TNF-α therapy is discontinued, patients should be monitored clinically and by MRI since relapse occurs in 50% of cases, particularly during the first year following therapy withdrawal (9, 48) and typically within the same neurological localization (9, 27). The reintroduction of anti-TNF-α therapy resulted in a favorable outcome in 100% of patients. Adverse events are common but rarely require permanent discontinuation. Infections are the most important adverse effects, accounting for approximately one-third of cases, but only one death related to unspecified infectious disease was reported in the literature (24). The risk of infectious complications is higher in patients already treated for a longer duration with corticosteroids and immunosuppressive therapy before the introduction of TNF-α antagonist therapy (21). These results highlight the benefits of TNF-α inhibitors in neurosarcoidosis and suggest that they should be prescribed earlier in the disease course. However, these drugs are currently not licensed nor reimbursed by the Belgian healthcare system for treating neurosarcoidosis.

B cell-Targeted therapy (rituximab) seems to have some efficacy in sarcoidosis especially in systemic sarcoidosis and even in neurosarcoidosis. However, this is based on small cohort studies, and there is insufficient data to support the use of rituximab over TNF inhibitors (47, 60).

Janus Kinase inhibitors (Jak inhibitors) are new drugs targeting the JAK/STAT pathways and are used in several diseases such as rheumatoid arthritis, inflammatory bowel disease, graft vs. host disease, and hemophagocytic lymphohistiocytosis (61). JAK/STAT plays a key role in the signaling pathways of several pro-inflammatory cytokines and thus may be a good therapeutic option. Tofacitinib and baricitinib have been used in refractory cutaneous and systemic sarcoidosis but data on neurosarcoidosis are lacking (62, 63).

### Outcome

In our cohort, favorable outcome was reported in up to 81% of patients, compared to 65% of cases in the literature. The higher favorable outcome in our cohort could be attributed to several factors. First, the majority of our patients were diagnosed between 2015 and 2021 and have therefore benefited from the most recent treatment strategies. Second, our cohort did not include patients with hydrocephalus which is known to have a worse outcome (7, 28). Despite the large proportion of favorable outcomes at the last follow-up, ~70% of patients experienced relapse and/or progression during their disease course. Moreover, some patients, even in case of stable inactive disease or remission, will experience a significant loss of autonomy due to neurological sequelae, especially in the case of spinal cord involvement (19, 53).

### Limitations

Our study has several limitations. First, our study and most studies included in the literature search were retrospective in nature with inherent limitations and were performed in tertiary centers leading possibly to selection bias. To maximize case ascertainment, we carefully and systematically reviewed all patients' medical records of our cohort and all available patient data from studies included in the literature review. Although possible neurosarcoidosis cases were not excluded in our study, as recommended since 2018 (13), it allowed us to include a larger number of patients and better reflect daily clinical practice. Second, we did not perform a systematic review. However, the scope and depth of our manuscript are extensive enough to render a review piece. Third, pooled analysis of literature data must be interpreted with caution due to the heterogeneity of inclusion criteria, neurological manifestations, treatment outcome definition, immunosuppressive therapy strategies, and their evolution over time, as well as the possible inclusion of some patients two times despite rigorous review of each study by the authors, inclusion date and centers, and contact of several corresponding authors. Moreover, all items were not reported for every patient. To compensate for this bias, results were presented as the percentage of patients for which the data were available [n/N (%)].

## Conclusion

Sarcoidosis is the most common non-infectious granulomatous disease affecting the nervous system. Its diagnosis remains challenging due to heterogeneity in clinical presentation and results of ancillary investigations. The results of our cohort and literature review provide relevant results regarding treatment with TNF-α antagonists and confirm their effectiveness in neurosarcoidosis. Additional studies, in particular multicenter clinical trials designed for rare diseases (64), are needed to confirm their safety, efficacy, and potential earlier place in the therapeutic armamentarium of neurosarcoidosis, as well as to determine the duration, tapering, and timing for the eventual interruption.

## Data availability statement

The original contributions presented in the study are included in the article/Supplementary material, further inquiries can be directed to the corresponding author/s.

## Ethics statement

The studies involving human participants were reviewed and approved by Comité d'Éthique Hospitalo-Facultaire (CEHF). Written informed consent for participation was not required for this study in accordance with the national legislation and the institutional requirements.

## Author contributions

PS, HY, and VP designed the study, take care of the patients, and wrote the manuscript. AK, LP, and AS corrected the manuscript and helped in the management of the patients. OG corrected the manuscript and helped with the interpretation of the radiological exam. All authors contributed to the article and approved the submitted version.

## Conflict of interest

The authors declare that the research was conducted in the absence of any commercial or financial relationships that could be construed as a potential conflict of interest.

## Publisher's note

All claims expressed in this article are solely those of the authors and do not necessarily represent those of their affiliated organizations, or those of the publisher, the editors and the reviewers. Any product that may be evaluated in this article, or claim that may be made by its manufacturer, is not guaranteed or endorsed by the publisher.
